# Cardiovascular disease: The Brazilian research contribution

**DOI:** 10.21470/1678-9741-2019-0285

**Published:** 2019

**Authors:** Erwin Krauskopf

**Affiliations:** 1 Universidad Andrés Bello - Facultad de Ciencias de la Vida, Santiago, Chile.; 2 Fundación Ciencia Para la Vida, Santiago, Chile.

Cardiovascular disease is the leading cause of death around the world. According to a study from the World Economic Forum, the economic burden of this disease to society reached US$ 863 billion in 2010, with an estimation to rise by 22% to US$ 1,044 in 2030^[1]^. Likewise, recent studies have shown that in Brazil ischemic heart disease and stroke have been the main cause of death since the end of the 1960s, costing a total of R$ 56.2 billion just in 2015^[2,3]^. Due to Brazil’s large size, its 27 states have developed unevenly, so states located in the south and southeast regions of the country are more developed and have the best infrastructure^[2]^. Hence, such differences ought to be considered when allocating resources efficiently to improve healthcare among the population. It is imperative to seek knowledge through locally-based research as its outcomes may be used as a tool to instruct policy makers, regional-level physicians, health professionals and the general population^[4]^.

To establish the Brazilian contribution to cardiovascular disease research, the Scival platform (www.scival.com) was used, which analyzes data from several sources such as Scopus and ScienceDirect. In the case of patent article citations, data emanate from European Patent Office, Intellectual Property Office, Japan Patent Office, United States Patent and Trademark Office and the World Intellectual Property Organization. A query was made to retrieve data from Brazil which had been published within the most recent 5-year period (2014-2018) in the field of “Cardiology and Cardiovascular Medicine”. One of the key features of Scival is that it disaggregates each field into specific research topics. As approximately 96,000 specific research topics have been defined, topic clusters are formed by aggregating topics with similar research interest, creating a broader area of research^[5]^. It is important to note that a publication can belong to only one topic, consequently, to one topic cluster.

The indicators used for this analysis were the following:

- Scholarly output: The number of documents published within the 5-year period in the topic cluster.
- Growth (%): This indicator represents the increase or decrease of published documents within the specific topic cluster in the 5-year period.
- International collaboration (%): The proportion of published documents authored by researchers from Brazil and another countries.
- Field-Weighted Citation Impact (FWCI): Indicator that refers to citations received in the year of publication plus the following 3 years. FWCI of 1.00 means that the publications have been cited at world average for similar publications. Thus, a score of 1.17 indicates that the outputs have been cited 17% more than expected. Contrarily, a FWCI of 0.77 means 23% less cited than the world average.
- Patent-cited scholarly output: The count of scholarly outputs published by the country that have been cited in patents.

As Table 1 reveals, the field of “Cardiology and Cardiovascular Medicine” in Brazil is constituted by 47 topic clusters, ordered according to the number of papers published within the 5-year period. The most prolific topic was “Percutaneous coronary intervention; Patients; Myocardial infarction”, with 584 papers, of which 49.3% were the product of an international collaboration. While its growth has diminished slightly over the 5-year period, its FWCI score denotes that these papers have been cited 99% more than expected. In fact, the six most prolific topic cluster exhibits an FWCI >1, revealing the quality of the work published.

**Table 1:** Topic clusters associated to the field of cardiovascular disease.

Topic cluster	Scholarly output	Growth (%)	International collaboration (%)	Field-Weighted Citation Impact
Percutaneous Coronary Intervention; Patients; Myocardial Infarction	584	-1.7	49.3	2
Atrial Fibrillation; Patients; Catheter Ablation	570	54.3	48.8	1.42
Cholesterol; Lipids; Atherosclerosis	492	15.8	35.8	2.71
Sepsis; Acute Kidney Injury; Patients	483	0.3	32.7	2
Anticoagulants; Patients; Venous Thromboembolism	472	28.1	31.4	1.25
Heart Rate; Blood Pressure; Patients	412	27.6	40.3	0.9
Wounds and Injuries; Pressure Ulcer; Bandages	385	60.6	12.7	0.56
Renin-Angiotensin System; Peptidyl-Dipeptidase A; Angiotensins	348	-7.4	36.8	0.91
Hypertension; Blood Pressure; Patients	337	6.2	35.6	2.41
Stroke; Patients; Cerebral Hemorrhage	303	4.6	30.7	1.09
Heart Failure; Patients; Brain Natriuretic Peptide	301	-6.5	36.2	1.76
Coronary Artery Disease; Patients; Echocardiography	264	19.3	45.8	1.07
Aortic Valve; Mitral Valve; Aortic Valve Stenosis	253	7.3	37.2	1.12
Ischemic Preconditioning; Reperfusion Injury; Ischemic Postconditioning	221	6.5	13.6	1.01
Heart Diseases; Patients; Congenital Heart Defects	211	-30.4	21.8	0.56
Abdominal Aortic Aneurysm; Aneurysm; Dissection	191	-2.6	22.0	0.47
Vasodilation; Endothelium; Dilatation	171	51.2	16.4	0.62
Catheters; Renal Dialysis; Central Venous Catheters	160	96.3	13.1	0.47
Pneumothorax; Lung; Pleural Effusion	142	11.6	22.5	0.59
Pulmonary Hypertension; Pulmonary Artery; Patients	136	61.2	47.4	1.11
Rehabilitation; Depression; Patients	135	63.2	38.5	0.83
Heart Arrest; Cardiopulmonary Resuscitation; Out-Of-Hospital Cardiac Arrest	115	23.6	41.2	1.01
Peripheral Arterial Disease; Ischemia; Extremities	113	-28.9	21.2	1.42
Vascular Stiffness; Pulse Wave Analysis; Blood Pressure	109	11.4	33.0	0.93
Matrix Metalloproteinases; Matrix Metalloproteinase 9; Metalloproteases	109	-22.8	33.0	0.7
Calcium; Calcium Signaling; Ion Channels	97	-6.1	53.6	0.83
Uric Acid; Gout; Hyperuricemia	95	20.7	33.7	1.26
Lymphedema; Breast Neoplasms; Chylothorax	90	4.2	15.6	0.51
Enterovirus; Poliomyelitis; Myocarditis	81	30.5	43.2	1.5
Endocarditis; Aneurysm; Patients	81	22.2	18.5	1.13
Heart-Assist Devices; Extracorporeal Membrane Oxygenation; Patients	78	-10.8	20.5	0.56
Erythrocyte Indices; Neutrophils; Lymphocytes	65	-25.8	10.8	0.52
Hypertrophic Cardiomyopathy; Myosins; Cardiomyopathies	61	21.2	32.8	0.7
Anthracyclines; Doxorubicin; Neoplasms	57	30.7	28.1	1.08
Sarcoidosis; Granuloma; Patients	52	119.4	23.1	0.6
Trachea; Thoracic Aorta; Bronchoscopy	50	47.7	16.0	0.92
Ischemia; Patients; Superior Mesenteric Artery	46	-0.4	17.4	0.68
Coronary Vessels; Pulmonary Artery; Fistula	39	-1.5	7.7	0.14
Heart Neoplasms; Myxoma; Echocardiography	38	131.2	26.3	1.27
Erythrocytes; Contrast Media; Blood	37	-24	21.6	0.81
Renal Artery; Renal Artery Obstruction; Hypertension	36	25.7	5.6	0.5
Hyponatremia; Sodium; Hyperkalemia	32	132.7	34.4	0.94
Inferior Vena Cava; Renal Veins; Syndrome	32	177.5	9.4	0.56
Heart; Acoustic Waves; Cardiology	30	-13.4	26.7	0.4
Pregnancy; Dissection; Coronary Vessels	27	-72.4	14.8	0.33
Pericarditis; Pericardial Effusion; Constrictive Pericarditis	22	76.1	9.1	0.32
Takotsubo Cardiomyopathy; Patients; Electrocardiography	17	-7.5	35.3	0.66

Another important aspect to consider is the impact of the Brazilian papers in patent generation. As Collins and Wyatt^[6]^ stated, the main characteristic of papers cited by patents must be in a rapidly developing field with a high scientific content. To understand the real value of these papers, 509 US patents issued from 1987 to 2003 cited 273 Chilean papers from several disciplines^[7]^. In this regard, Table 2 summarizes the Brazilian papers cited in 16 patents issued from 2014 to 2018.

**Table 2:** Brazilian papers cited in patents.

Title	Year	Source	Volume	Pages
Pleural subxyphoid drain confers better pulmonary function and clinical outcomes in chronic obstructive pulmonary disease after off-pump coronary artery bypass grafting: A randomized controlled trial	2014	Brazilian Journal of Cardiovascular Surgery	29	588-594
Butyrate impairs atherogenesis by reducing plaque inflammation and vulnerability and decreasing NFκB activation	2014	Nutrition, Metabolism and Cardiovascular Diseases	24	606-613
Vorapaxar in acute coronary syndrome patients undergoing coronary artery bypass graft surgery: Subgroup analysis from the TRACER trial (Thrombin Receptor Antagonist for Clinical Event Reduction in Acute Coronary Syndrome)	2014	Journal of the American College of Cardiology	63	1048-1057
Statin-associated muscle symptoms: impact on statin therapy - European Atherosclerosis Society Consensus Panel Statement on Assessment, Aetiology and Management	2015	European Heart Journal	36	1012-1022
Management of pulmonary arterial hypertension	2015	Journal of the American College of Cardiology	65	1976-1997
Simplified Method for Vagal Effect Evaluation in Cardiac Ablation and Electrophysiological Procedures	2015	JACC: Clinical Electrophysiology	1	451-460
2017 HRS/EHRA/ECAS/APHRS/SOLAECE expert consensus statement on catheter and surgical ablation of atrial fibrillation: Executive summary	2017	Heart Rhythm	14	e445-e494
Targeting PCSK9 for therapeutic gains: Have we addressed all the concerns?	2016	Atherosclerosis	248	62-75
Ticagrelor for Prevention of Ischemic Events After Myocardial Infarction in Patients with Peripheral Artery Disease	2016	Journal of the American College of Cardiology	67	2719-2728
Association of endothelial dysfunction with cardiovascular risk factors and new-onset diabetes mellitus in patients with hypertension	2018	Journal of Clinical Hypertension	20	935-941

Undoubtedly, research developed by Brazilian investigators in topics related to cardiovascular disease has proven valuable, not only for the academic community, but also for the industry. I believe now is the time to make this research useful for policy makers to influence their assessments.
